# Supplementary material for: Autism-associated SHANK3 missense point mutations impact conformational fluctuations and protein turnover at synapses
Source: eLife. 2021 May 4;10:e66165. doi: 10.7554/eLife.66165 (PMC8169116; doi:10.7554/eLife.66165)
Supplement: Transparent reporting form [file elife-66165-transrepform.docx]

***eLife’s* transparent reporting form**

We encourage authors to provide detailed information *within their submission* to facilitate the interpretation and replication of experiments. Authors can upload supporting documentation to indicate the use of appropriate reporting guidelines for health-related research (see [EQUATOR Network](http://www.equator-network.org/%20)), life science research (see the [BioSharing Information Resource](https://biosharing.org/)), or the [ARRIVE guidelines](http://www.plosbiology.org/article/info:doi/10.1371/journal.pbio.1000412) for reporting work involving animal research. Where applicable, authors should refer to any relevant reporting standards documents in this form.

If you have any questions, please consult our Journal Policies and/or contact us: [editorial@elifesciences.org](mailto:editorial@elifesciences.org).

**Sample-size estimation**

- You should state whether an appropriate sample size was computed when the study was being designed
- You should state the statistical method of sample size computation and any required assumptions
- If no explicit power analysis was used, you should describe how you decided what sample (replicate) size (number) to use

Please outline where this information can be found within the submission (e.g., sections or figure legends), or explain why this information doesn’t apply to your submission:

Figure 5, Figure 7 and Figure 7 - figure supplement 1: The simple size was estimated for experiments in neurons using common standards of the field (at least two independent primary culture preparations).
Figure 5: between 7 - 39 cells per condition depending on the panel (specific numbers indicated in the figure legend)

Figure 7: between 19 - 60 spines per condition

Figure 7 - figure supplement 1: 11 - 16 cells per condition

**Replicates**

- You should report how often each experiment was performed
- You should include a definition of biological versus technical replication
- The data obtained should be provided and sufficient information should be provided to indicate the number of independent biological and/or technical replicates
- If you encountered any outliers, you should describe how these were handled
- Criteria for exclusion/inclusion of data should be clearly stated
- High-throughput sequence data should be uploaded before submission, with a private link for reviewers provided (these are available from both GEO and ArrayExpress)

Please outline where this information can be found within the submission (e.g., sections or figure legends), or explain why this information doesn’t apply to your submission:

Information regarding replicas can be found in figure legends and methods. For cellular data 2-4 independent preparations of primary neurons were used, several cells were analyzed per preparation and condition. Few cells were excluded prior to analysis based on morphological features and criteria (specifically, unhealthy cells showing varicosities or membrane ruptures). For individual measurements of protein turnover using FRAP several individual synapses were measured on the same cell. Statistical information and corresponding p values as well as the number of independent culture preparations, cells and synapses are given in the figure legend (Figure 5, Figure 7, Figure 7 - figure supplement 1).

For Figure 6, 3-4 independent co-immunoprecipitation experiments were performed and quantified. Statistical information and corresponding p values as well as the number of independent experiments are given in the figure legend.

**Statistical reporting**

- Statistical analysis methods should be described and justified
- Raw data should be presented in figures whenever informative to do so (typically when N per group is less than 10)
- For each experiment, you should identify the statistical tests used, exact values of N, definitions of center, methods of multiple test correction, and dispersion and precision measures (e.g., mean, median, SD, SEM, confidence intervals; and, for the major substantive results, a measure of effect size (e.g., Pearson's r, Cohen's d)
- Report exact p-values wherever possible alongside the summary statistics and 95% confidence intervals. These should be reported for all key questions and not only when the p-value is less than 0.05.

Please outline where this information can be found within the submission (e.g., sections or figure legends), or explain why this information doesn’t apply to your submission:

Statistical analysis was done using OriginPro 2019b. For each dataset a normality test was performed initially. For normally distributed data, a two-way ANOVA with Tukey post-hoc test was calculated (Figure 7 - figure supplement 1). For Figure 5 and 6, a one-way ANOVA with Bonferroni multiple comparisons correction was done for normally distributed data.

For not normally distributed data, a Kruskal-Wallis-ANOVA with post-hoc test was calculated. Statistical parameters (p-values, number of individual measurements, number of biological replicas, means, SEM, P, Pearson’s r, reduced chi-squared) are given either in the figures themselves or in the corresponding figure legend or Supplementary File 1

(For large datasets, or papers with a very large number of statistical tests, you may upload a single table file with tests, Ns, etc., with reference to sections in the manuscript.)

**Group allocation**

- Indicate how samples were allocated into experimental groups (in the case of clinical studies, please specify allocation to treatment method); if randomization was used, please also state if restricted randomization was applied
- Indicate if masking was used during group allocation, data collection and/or data analysis

Please outline where this information can be found within the submission (e.g., sections or figure legends), or explain why this information doesn’t apply to your submission:

Part of the confocal fluorescence data shown in Figure 5 were coded and analyzed blindly. Appropriate controls were always included and measured in parallel to experimental groups.

**Additional data files (“source data”)**

- We encourage you to upload relevant additional data files, such as numerical data that are represented as a graph in a figure, or as a summary table
- Where provided, these should be in the most useful format, and they can be uploaded as “Source data” files linked to a main figure or table
- Include model definition files including the full list of parameters used
- Include code used for data analysis (e.g., R, MatLab)
- Avoid stating that data files are “available upon request”

Please indicate the figures or tables for which source data files have been provided:

We provide videos of computed 1000ns MD trajectories for each protein (WT and mutants), related to Figure 4 as well as Figure 1 - figure supplement 1.
